# Modeling Left Atrial Flow, Energy, Blood Heating Distribution in Response to Catheter Ablation Therapy

**DOI:** 10.3389/fphys.2018.01757

**Published:** 2018-12-14

**Authors:** Desmond Dillon-Murphy, David Marlevi, Bram Ruijsink, Ahmed Qureshi, Henry Chubb, Eric Kerfoot, Mark O'Neill, David Nordsletten, Oleg Aslanidi, Adelaide de Vecchi

**Affiliations:** ^1^School of Biomedical Engineering and Imaging Sciences, King's College London, London, United Kingdom; ^2^School of Engineering Sciences in Chemistry, Biotechnology and Health, KTH Royal Institute of Technology, Stockholm, Sweden; ^3^Department of Cardiothoracic Surgery, Stanford University, Palo Alto, CA, United States

**Keywords:** left atrium, computational fluid dynamics, atrial fibrillation, thermal modeling, catheter ablation

## Abstract

**Introduction:** Atrial fibrillation (AF) is a widespread cardiac arrhythmia that commonly affects the left atrium (LA), causing it to quiver instead of contracting effectively. This behavior is triggered by abnormal electrical impulses at a specific site in the atrial wall. Catheter ablation (CA) treatment consists of isolating this driver site by burning the surrounding tissue to restore sinus rhythm (SR). However, evidence suggests that CA can concur to the formation of blood clots by promoting coagulation near the heat source and in regions with low flow velocity and blood stagnation.

**Methods:** A patient-specific modeling workflow was created and applied to simulate thermal-fluid dynamics in two patients pre- and post-CA. Each model was personalized based on pre- and post-CA imaging datasets. The wall motion and anatomy were derived from SSFP Cine MRI data, while the trans-valvular flow was based on Doppler ultrasound data. The temperature distribution in the blood was modeled using a modified Pennes bioheat equation implemented in a finite-element based Navier-Stokes solver. Blood particles were also classified based on their residence time in the LA using a particle-tracking algorithm.

**Results:** SR simulations showed multiple short-lived vortices with an average blood velocity of 0.2-0.22 m/s. In contrast, AF patients presented a slower vortex and stagnant flow in the LA appendage, with the average blood velocity reduced to 0.08–0.14 m/s. Restoration of SR also increased the blood kinetic energy and the viscous dissipation due to the presence of multiple vortices. Particle tracking showed a dramatic decrease in the percentage of blood remaining in the LA for longer than one cycle after CA (65.9 vs. 43.3% in patient A and 62.2 vs. 54.8% in patient B). Maximum temperatures of 76° and 58°C were observed when CA was performed near the appendage and in a pulmonary vein, respectively.

**Conclusion:** This computational study presents novel models to elucidate relations between catheter temperature, patient-specific atrial anatomy and blood velocity, and predict how they change from SR to AF. The models can quantify blood flow in critical regions, including residence times and temperature distribution for different catheter positions, providing a basis for quantifying stroke risks.

## 1. Background

Atrial Fibrillation (AF) is the most common cardiac arrhythmia, affecting over 30 million people worldwide (Kirchhof et al., 2016). It is characterized by irregular, rapid activations of the atria and carries a high risk of heart failure and stroke. This irregular electrical activity causes the atrium to quiver instead of contracting, thus compromising the atrial flow dynamics and the amount of blood pushed into the ventricle. In the case of the Left Atrium (LA), this can reduce cardiac output by up to 30% (Iwasaki et al., 2011). Catheter Ablation (CA) has proven to be an effective treatment for permanent termination of AF and is gradually supplanting antiarrhythmic drug therapy. This procedure involves the targeted application of Radio-Frequency (RF) energy to the myocardium to create transmural lesions that neutralize the abnormal electrical impulses by isolating the source of irregular activity (e.g., thepulmonary veins (PV), the mitral annulus and isthmus) from the surrounding tissue and restore the normal Sinus Rhythm (SR) (O'Neill et al., 2007).

The increased risk of heart failure and stroke in AF patients have been associated with higher residence times of blood in the fibrillating LA (Camm et al., 2012; Soor et al., 2016). In the healthy atria, blood forms high-speed vortices, while in AF these structures merge into a single slow-moving vortex, contributing to blood stasis and potential thromboembolism formation (Fyrenius, 2001; Fluckiger et al., 2013; Koizumi et al., 2015; Gülan et al., 2017). Such effects of AF on the atrial flow dynamics become even more important during CA. Thus, the risk of thrombus formation, which is high in regions with propensity to blood stasis such as the LA appendage, is exacerbated by both the low blood flow velocities that occur in AF and the localized heating of the blood surrounding the site where CA is performed (Cibis et al., 2017). Recent studies showed that the blood washout in the LA is considerably lower during fibrillation and the number of blood particles remaining in the LA appendage after three cycles almost doubles in AF compared to SR conditions, suggesting an increased likelihood of blood stasis and subsequent thrombus formation (Masci et al., 2017a,b). A 10% incidence of thrombus was reported during CA (Ren et al., 2004). A study in dogs showed a rate of thromboembolism 2.5 times higher after CA (mean temperature 66 ± 5.5°C) than after cryoenergy ablation (mean temperature −60 ± 12.1°C) (Khairy et al., 2003). *In-vitro* evidence proved that plasma aggregation occurs in a few seconds at 50–80°C in stationary blood (Demolin et al., 2002). In patients, an incidence of coagulum up to 8% was found for CA temperatures above 75°C (Calkins et al., 1994).

The interplay between atrial flow, catheter temperatures and thrombogenic events during AF is poorly understood, due to the difficulty in characterizing atrial flow dynamics and to the scarcity of data on blood residence times in the LA pre- and post-CA. As blood flow rates vary considerably inside the LA, the temperature distribution and heat dissipation are hard to predict. Although various models can successfully quantify atrial blood flow in AF, none of these includes the simulation of CA and temperature in the blood flow to address this issue (Mouret et al., 2004; Zhang and Gay, 2008; Chnafa et al., 2012, 2014; Koizumi et al., 2015; Vedula et al., 2015). Conversely, tissue temperature distribution during CA has been simulated in a variety of models that either do not include blood flow (Tungjitkusolmun et al., 2001; Lai et al., 2004; Gallagher et al., 2013), or only simulate the cooling effect of blood flow on the ablated tissue rather than the effect of heat on the blood itself (Jain and Wolf, 2000a,b; Berjano, 2006; González-Suárez and Berjano, 2016).

Blood residence times in the atria, and their links with thrombi formation and movement, have also been poorly characterized. Particle tracking provides a well-known technique to classify blood flow components based on their residence time in the heart. This type of analysis relies on the existence of a time point, such as the onset of systole in the Left Ventricle (LV), where there is no inflow nor outflow from the chamber, and from which the particle tracking is performed (Bolger et al., 2007; Eriksson et al., 2010). This approach was used to understand disease progression in the LV of patients with dilated cardiomyopathy (Carlhäll and Bolger, 2010; Eriksson et al., 2011, 2012). More recently, a similar technique was proposed to investigate atrial flow dynamics through continuous particle seeding from the Pulmonary Vein (PV), since blood inflow in the LA occurs continuously throughout both systole and diastole, unlike in the LV, with preliminary results showing that approximately half of the blood volume entering the healthy LA in one cycle is ejected in the same cycle (Gaeta et al., 2018).

The goal of this study is to understand better how LA flow is affected first by AF, then by AF termination by CA. Personalized models are generated by combining information from imaging data (Cine MRI and Doppler echocardiography) and atrial wall motion tracking. Computational fluid dynamics (CFD) with heat modeling is then performed using the Finite Element (FE) based software package CHeart (Lee et al., 2016; Hessenthaler et al., 2017) to simulate the patient-specific fluid-thermal dynamics, with further application of the particle tracking techniques. This workflow is applied to two patients presenting with AF pre- and post-CA, to explore how the complex fluid-thermal dynamics and flow distribution change between AF (pre-CA) and SR (post-CA).

## 2. Methods

### 2.1. Patient Data

SSFP Cine MRI data with a spatial resolution of 1.25 × 1.25 × 10 mm^3^ and temporal resolution of 50 timesteps/cycle were acquired for the full heart from two patients (Chubb et al., 2018). Data for patient A were acquired 84 days prior to and 89 days post CA. Data for patient B were acquired 63 days prior to and 90 days post CA. This patient also presented with type 2 diabetes mellitus. Both patients exhibited persistent AF in initial scans, and SR in the follow up scan. Patient A's was 69 BPM in the initial scan and 70 BPM in the post treatment scan, while Patient B's heart rate was 82 BPM for both scans. This data is summarized in Table 1.

**Table 1:** Patient data.

	**Age range**	**Vascular**	**AF duration**	**LA**	**LA max**	**LV**
	**(Years)**	**disease**	**(years)**	**fibrosis**	**Vol (ml)**	**EDV (ml)**
Patient A	55–60	Yes	2	Nil	113	114
Patient B	55–60	Nil	4	Nil	197	210
	**HR pre CA**	**HR post CA**	**LV stroke**	**LV stroke**		
	**BPM**	**BPM**	**Pre CA (ml)**	**Post CA (ml)**		
Patient A	69	70	56	80		
Patient B	82	82	101	149		

*A summary of the relevant data for the patients used in this study*.

### 2.2. Segmentation Process and Mesh Generation

**Figure 1:**
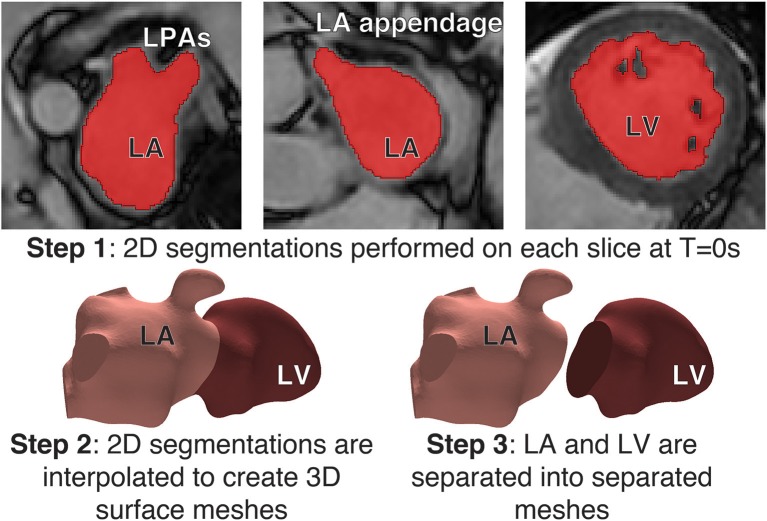
Segmentation process: Step 1: **(Top)** Segmentations of the LA and LV were performed with the image processing package MITK on each Cine MRI dataset at Time = 0s to create a patient-specific 3D atrial and ventricle mesh. Segmentations were performed on each slice in the axial plane. Step 2 **(Bottom-left)** A 3D interpolation is applied to the segmentation to create a smooth surface mesh. Step 3: **(Bottom-right)** the mesh is separated into the LA and LV at the mitral valve.

Segmentations of the LA and LV were performed with the image processing package MITK (Wolf et al., 2005) on each Cine MRI dataset at end-systole to create a patient-specific 3D atrial and ventricular mesh (Figure 1). Surface smoothing was applied via a 3D mathematical interpolation routine. Care was taken to insure the resulting interpolated segmentation conformed closely to the imaging data (Maleike et al., 2009). The atrial segmentation was processed using the software package Paraview (Ahrens et al., 2005) to identify the inflow planes for each of the PV and the outflow plane in the Mitral Valve (MV). A tetrahedral volume mesh of the LA was then generated from the smoothed surface mesh using the software package VMTK (Antiga et al., 2008) (*h*_*max*_ ≈ 2.9 mm, *h*_*min*_ ≈ 0.7 mm, and *h*_*mean*_ ≈ 1.5 mm) to perform FE flow simulations.

### 2.3. Motion Tracking

**Figure 2:**
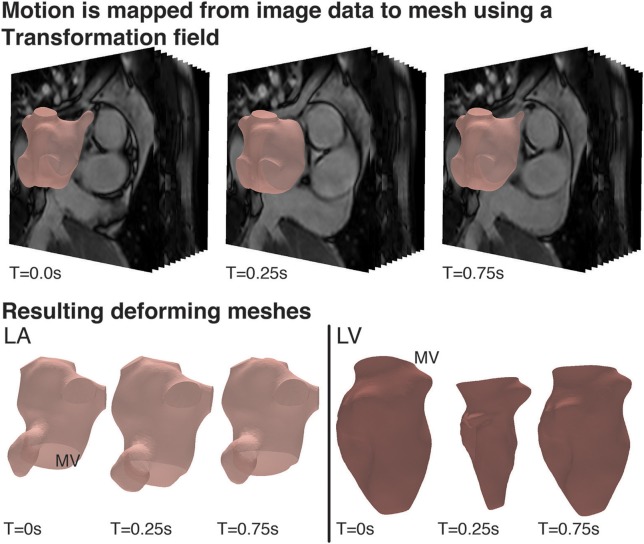
Cardiac Motion Tracking: **(Top)** Using the visualization and analysis package, Eidolon, an algorithm based on MIRTK which uses temporal-sparse free form deformations applied to the Cine MRI data to generate a transformation field for the entire image volume describing the motion of structures throughout the time-dependant image series, including the motion of the LA and LV. **(Bottom)** The tracking field was then applied to the LA and LV mesh and the deforming mesh was visually checked to ensure tracking was accurate throughout the cycle.

The visualization and analysis of the data was performed using the software package Eidolon (Kerfoot et al., 2016). Specifically, the motion of structures throughout the time-dependant image series was described via a motion tracking algorithm based on temporal sparse free-form deformations, where the deformation is reconstructed from an image sequence. A reference frame was chosen as source image for the other images in the sequence. This algorithm guarantees the periodicity of the deformation by enforcing cyclic motion. The algorithm was applied to the Cine MRI data to generate a transformation field for the entire image volume that represents the wall motion of the LA and LV. This tracking field was then applied to the LA and LV surfaces meshes. The deforming mesh was visually checked in three different views (sagittal, axial and coronal) to ensure tracking was accurate throughout the cycle. This process resulted in a deformed surface mesh for each of the 50 steps/cardiac cycle for both the LA and LV. Specifically, the motion of structures throughout the time-dependant image series was described via a motion tracking algorithm based on temporal sparse free-form deformations, where the deformation is reconstructed from an image sequence (Figure 2).

### 2.4. Boundary Conditions

The nodal coordinates of the 50 surface meshes of the LA were then interpolated with cubic splines and a periodic end condition to artificially increase the temporal resolution to 1,000 steps/cycle. A wall velocity boundary condition for each step was calculated and was then applied at the LA-blood interface to deform the mesh tracking the LA structure in the image throughout the cardiac cycle. The same steps were applied to the LV.

Using the tracked LV mesh, the volume of each chamber was calculated throughout the cardiac cycle. Flow through the MV, *Q*_*MV*_, was quantified by calculating the differential in positive volume change, dVdt from maximum to minimum volume over the cycle and assuming perfect functioning of the aortic valve and MV, such that

QMV=0, ifdVdt is negative,QMV=dVdt, if dVdt is positive.

The resulting flow velocity waveforms were validated against velocities recorded at the MV using Doppler ultrasound. The MV was defined as an ellipse fitted to the MV annulus. *Q*_*MV*_ was interpolated across this elliptical valve with a paraboloid shape to create a time-dependant velocity field, which was applied as a Dirichlet boundary condition on the MV plane. The maximum velocities were defined in the centre of the MV in a direction normal to the MV face and zero velocity was prescribed at the edges of the ellipse.

At each of the PV faces backflow stabilization, zero pressure stabilizing Neumann conditions were applied to avoid an excessive flux of momentum into the domain, and hence prevent instabilities, following a previously proposed approach (Esmaily Moghadam et al., 2011).

### 2.5. Computational Fluid Dynamics Simulations

CFD simulations of the patient-specific LA flow were then performed using the stabilized Streamline Upwind Petrov-Galerkin (SUPG) Arbitrary Lagrangian-Eulerian (ALE) Navier-Stokes solver for incompressible flows implemented in the software package CHeart (Lee et al., 2016). The temporal resolution was set to 0.73 ms, resulting in 1000 time steps per cycle. Up to 10 cardiac cycles were simulated using 98 cores of a 640 core SGI Altix-UV high performance computing (HPC) cluster with Nehalem-EX architecture at King's College London. Blood density ρ was defined as 1.06 kg/m^3^ and viscosity μ as 3.5 × 10^−3^ Pa s. The results were checked to ensure periodicity.

Flow energies were also computed to describe LA function. Kinetic Energy (KE) is directly related to flow velocities and is described mathematically as

$$\begin{array}{l} KE=\frac{\rho }{2}∥v_{blood}{∥}^{2} \end{array}$$

where *v*_*blood*_ is the blood velocity (m/s).

Viscous energy quantifies the energy dissipation that occurs due to the conversion of KE to thermal energy or to the presence of high-friction, vortical flow. The rate of viscous energy dissipation, ($\dot{E}$), can be described as

$$\begin{array}{l} Ė=\mu \frac{{\left[\nabla v_{blood}+{\left(\nabla v_{blood}\right)}^{T}\right]}^{2}}{2} \end{array}$$

To compare the changes in *KE* and $\dot{E}$ between the AF and SR states, the data was integrated over the volume of the atrial cavity (Elbaz et al., 2017). Reduced vortical flows have been associated with patients in AF and are associated with an increased risk of thrombus formation and embolization (Fyrenius, 2001).

### 2.6. Particle Tracking

Following the CFD simulations particle tracking was applied to the generated LA velocity field for the final simulated cycle. The geometry was uniformly seeded with massless particles at the instant the MV opens. The seeding was performed at the onset of systole in all cases. A uniform grid was imposed on the entire LA cavity and the particles were seeded at each nodal position of the grid from this starting point (no reseeding was performed). The number of particle was approximately 10,000 with small variations between the two patient cases due to differences in the size of the cavity. The trajectory of each particle was computed using 4th order Runge-Kutta estimations based on the velocity field for a single full cardiac cycle and as such was unique. Hence, in order to cover the entire cycle, each particle was tracked backwards and forwards in time starting from a fixed seeding point at the onset of systole, so the forward tracing would cover systole and the backward tracing diastole.

Particles were defined by their behavior during the cycle, in analogy with the spatial flow component analysis proposed in previous studies on left ventricular fluid dynamics (Bolger et al., 2007; Eriksson et al., 2010). Retained Inflow (RI) was defined as those particles that enter via the pulmonary veins (PV) during diastole and are not ejected at the end of the cycle analyzed. Delayed Ejection (DE) was defined as those particles present in the LA at the start of the cycle (diastole) and ejected during systole. Residual volume (RV) was defined as that flow that resides in the LA for at least two complete cycles. Finally diastolic Direct Flow (DF) was defined as the blood that enters the LA during diastole and is ejected in the following systole. Particles were also tagged based on which PV they emanated from to allow quantification of the contribution of each Pressure-Volume (PV).

Particle tracking convergence analysis was performed with respect to time tracking and particle density in a previous cardiac dataset (De Vecchi et al., 2018). These indicated that stabilized flow components (RI, DE, RV, DF) were achieved with ~10,000 uniformly seeded particles, traced forward and backward with 0.5 ms time stepping.

### 2.7. Thermal Simulations

Thermal processes during CA were modeled using a modified version of the Pennes bioheat equation (Soor et al., 2016):

$$\begin{array}{l} Ṫ+\left(v_{blood}-v_{wall}\right)\cdot \nabla T-\nabla \cdot D\nabla T-Q_{cath}+Q_{perf}-Q_{meta}=0 \end{array}$$

The heat loss due to perfusion (*Q*_*perf*_) and metabolic heat gain (*Q*_*meta*_) were set to zero, since they have negligible physiological values. *v*_*wall*_ is wall velocity m/s calculated from the Computational Fluid Dynamics (CFD) simulation. The heat gain from the catheter (*Q*_*cath*_) was defined in a sphere of radius 5 mm near the LA wall, which mimiced the catheter tip; the value of *Q*_*cath*_ was chosen to produce temperature of 90°C at the tip (Dillon-Murphy et al., 2017). *T* is the temperature of the blood in °C. *D* = ^*k*^/_ρ·*c*_, *k* being the thermal conductivity of blood set to 0.57 W/m·K and *c* is the blood specific heat capacity, which was set to 3.9 J/kg·K. The initial conditions for blood temperature was 37°C everywhere except for the catheter tip. Zero-flux boundary conditions on temperature were applied at all boundaries.

**Figure 3:**
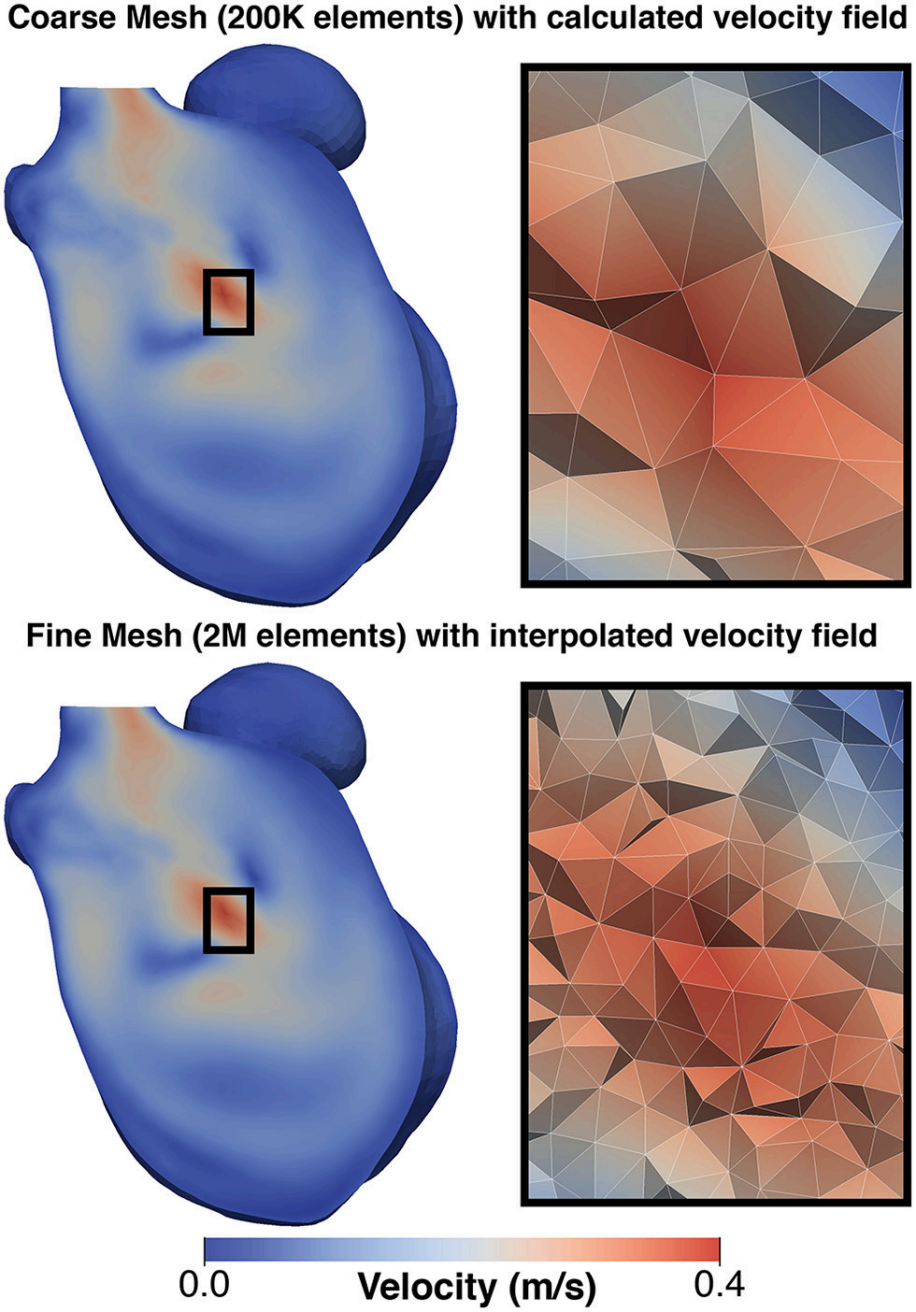
Comparison of mesh for CFD simulation and thermal simulation: **(Top)** shows a the velocity field generated on the CFD and **(Bottom)** shows the same field mapped to the finer mesh used for the thermal simulation. To the left of each image is a detailed view of the mesh structure in the center of the domain. This mapping was performed to avoid numerical issues due to the adjective dominant behavior of heat in blood.

The thermal problem was found to be dominated by advection, which was reflected in a high Peclet number (~10^7^). Therefore, the simulation of the thermal diffusion on the CFD mesh resulted in numerical instabilities. To resolve this, the velocity field from the CFD analysis was interpolated and mapped to a denser LA mesh (*h*_*max*_ ≈ 1.75 mm, *h*_*min*_ ≈ 0.38 mm, and *h*_*mean*_ ≈ 0.92 mm) (Figure 3). Simulations on such a fine mesh with about 2 million elements were less efficient computationally, but allowed us to avoid instabilities and generate the required proof-of-concept data.

## 3. Results

### 3.1. Flow Velocity Analysis

Both simulations of SR patients showed flow velocities within a normal physiological range. Patient A ranged between 0 and 2.0 m/s, mean velocity 0.22 m/s, while patient B ranged between 0 and 2.3 m/s with a mean velocity of 0.2 m/s with blood forming several fast, short-lived vortices in both cases (see Supplementary Video 1 for patient A).

Simulations of AF patients tended to show larger slower vortices in the middle of the LA cavity (see Supplementary Video 2 for patient A) compared to those in SR, and there was a greater degree of stagnant flow in the LA appendage of both patients. When in AF patient A exhibited velocities in the range 0–1.2 m/s, mean velocity 0.08 m/s and patient B exhibited velocities in the range of 0–1.5 m/s with mean velocity of 0.14 m/s, a drop of 64 and 30% from SR, respectively. These simulation results are in good agreement with previous patient measurements based on the application of 4D flow MRI and phase-contrast MRI (Fluckiger et al., 2013).

After treatment, when the patients were in SR, both patients exhibited a relatively normal double-peaked MV flow waveform, with the first peak corresponding to the main outflow wave and the second to the atrial contraction in late diastole.

**Figure 4:**
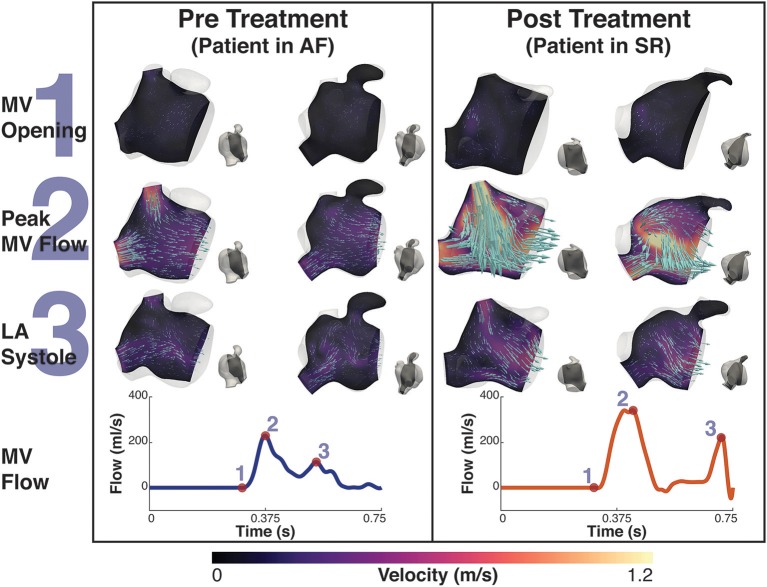
Patient A velocity simulation. The image shows two panels describing the velocity field for patient A. Both panels show the magnitude of velocity field with scaled arrows overlaid indicating the velocity direction for two 2D-planes positioned in the atrium. To the right of each velocity plot is a second view of the plane's location in the atrium at that instant. The left shows the velocity pre treatment in the fibrillating atria and the right panel the results post treatment. The bottom of each panel is the MV velocity calculated from the differential change in volume of the LV. The velocity is shown at three instants, 1- MV opening, just prior to the MV opening, 2- Peak MV Flow, the instant where there is maximal flow through the MV, and 3- LA systole, where there is an increase in MV flow from the contraction of the LA. These points are indicated on each flow waveform.

**Figure 5:**
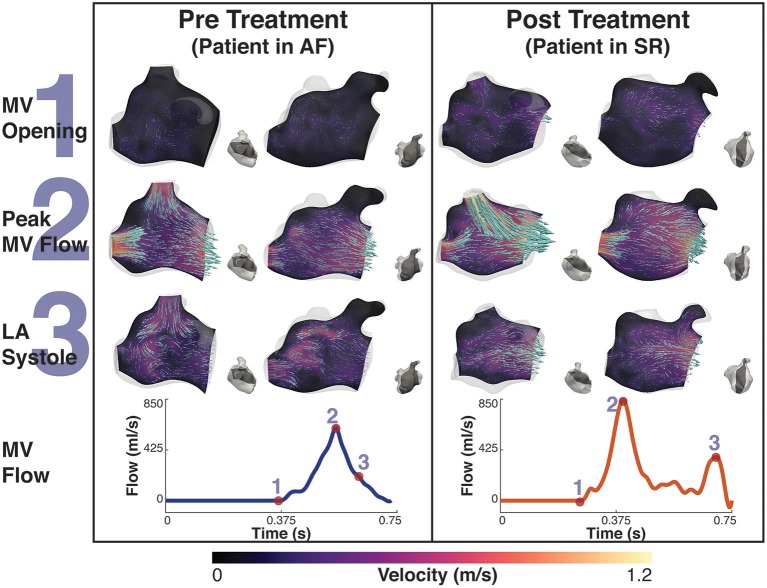
Patient B velocity simulation. The image shows two panels describing the velocity field for patient B. Both panels show the magnitude of velocity field with scaled arrows overlaid indicating the velocity direction for two 2D-planes positioned in the atrium. To the right of each velocity plot is a second view of the plane's location in the atrium at that instant. The left panel shows the velocity pre treatment in the fibrillating atria and the right panel the results post treatment. The bottom of each panel is the MV velocity calculated from the differential change in volume of the LV. The velocity is shown at three instants, 1- MV opening, just prior to the MV opening, 2- Peak MV Flow, the instant where there is maximal flow through the MV, and 3- LA systole, where there is an increase in MV flow from the contraction of the LA. These points are indicated on each flow waveform.

Before treatment, when the patients were in AF, the double-peaked MV flow was less evident. In patient A both outflow peak velocities were approximately 37 and 50% lower in AF than in SR (Figure 4). In patient B, while in AF, the two velocity peaks were fused into a single one that occurred later in systole and had a lower magnitude than the first main peak SR (25% reduction), as show in Figure 5.

When comparing AF to SR conditions in the Left Atrial Appendage (LAA), the velocity went from a maximum value of 0.22 m/s and a mean value of 0.02 m/s when in AF to a maximum value of 0.75 m/s and a mean value of 0.05 m/s when in SR for patient A, and a maximum value of 0.35 m/s and a mean value of 0.05 m/s to a maximum value of 0.44 m/s and a mean value of 0.06 m/s for patient B.

### 3.2. Energy Analysis

**Figure 6:**
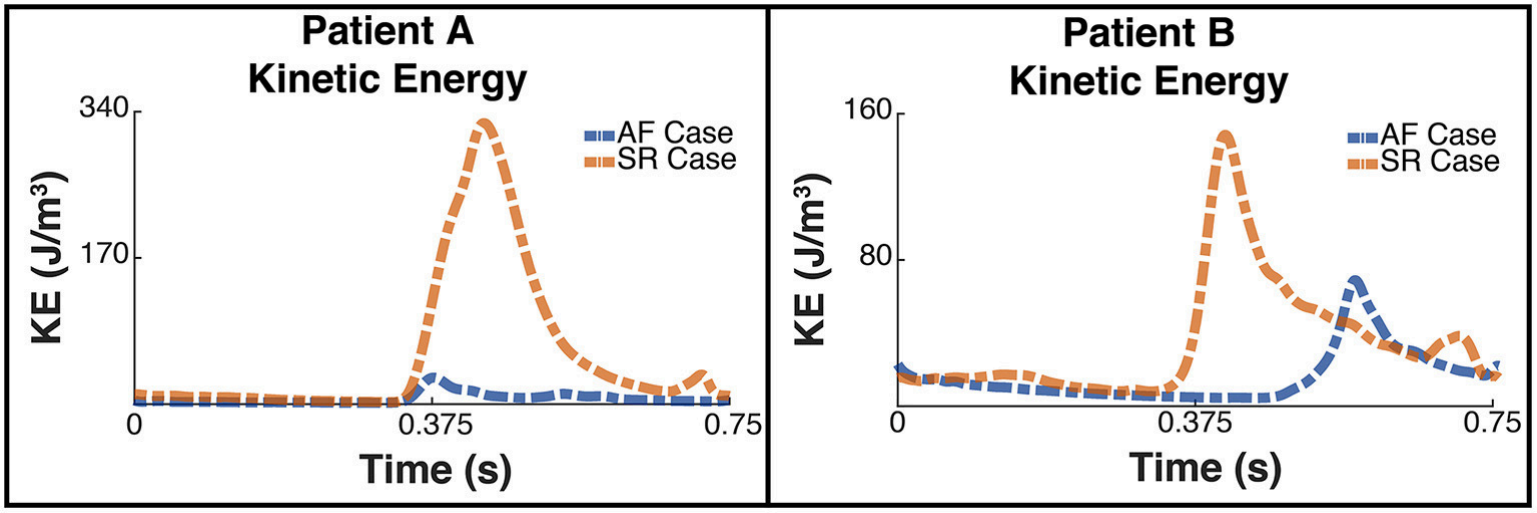
Total Kinetic Energy. The image shows two panes of the total kinetic energy which is proportional to $∥v_{blood}{∥}^{2}$ for patient A **(Left)** and patient B **(Right)**. The blue dashed line shows the total kinetic energy for each patient pre ablation, ie when the patient is in AF, and the orange line shows the post treatment SR case.

Figure 6 shows the plot of the calculated kinetic energy for patient A and patient B for the cardiac cycle, which is proportional to $∥v_{blood}{∥}^{2}$. The dashed blue line shows the results for the AF case and the dashed orange shows the results for the SR case. Both patients showed a significant increase in the total kinetic energy in SR with the mean KE increasing 9-fold from 6.3 J/m^3^ when in AF to 58.8 J/m^3^ when in SR for patient A, and increasing over 2-fold from 15.9 J/m^3^ to 34.8 J/m^3^ for patient B.

**Figure 7:**
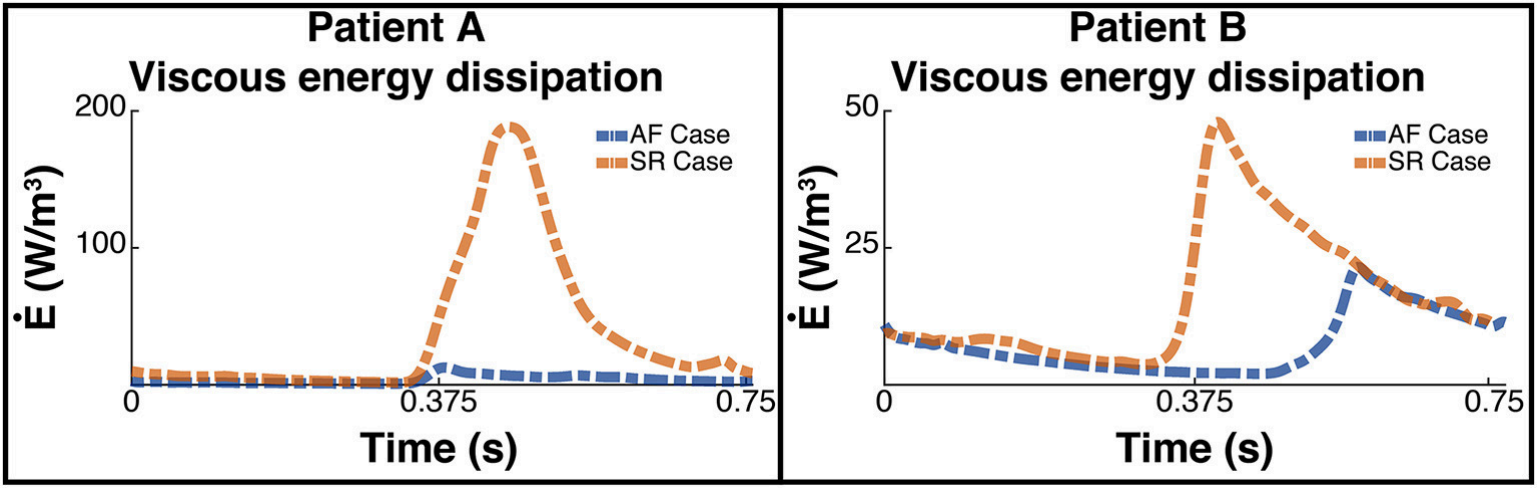
Rate of viscous energy dissipation. The image shows two panes of the viscous energy loss waveforms for patient A **(Left)** and patient B **(Right)**. The blue dashed line shows the instantaneous energy loss for each patient pre ablation, i.e., when the patient is in AF, and the orange line shows the post treatment SR case.

This increase in overall KE is associated with an increase in the rate of viscous energy loss as shown in Figure 7. The mean $\dot{E}$ for patient A increased 10-fold from 3.7 W/m^3^ to 38.3 W/m^3^, and for patient B increased over 2-fold from 7.4 to 16 W/m^3^.

The energy peaks in Figures 6, 7 show a large difference in magnitude between AF and SR, especially in patient A, supporting our observation that restoration of SR by CA can improve dramatically the intra-cavity flow velocity.

### 3.3. Particle Tracking

**Figure 8:**
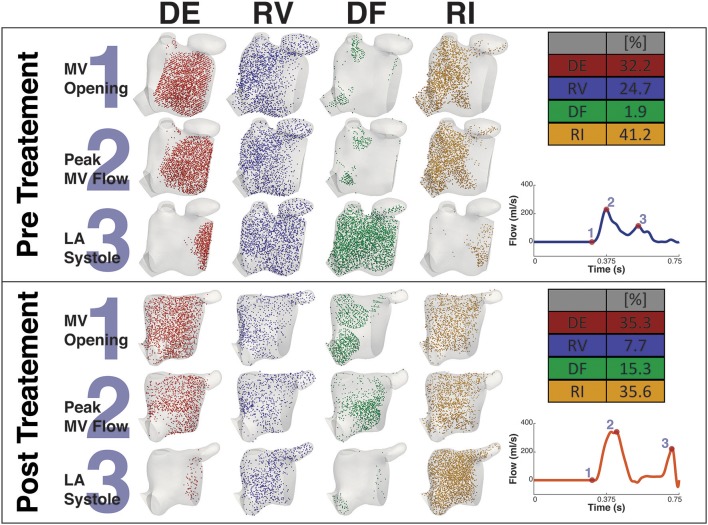
Particle tracking results Patient A. The image shows the particle tracking results for patient A. The image consists of two panels. Each panel shows the visualization of the different classification of particles DE, RV, DF, and RI at three points in the cardiac cycle, just prior to *MV Opening* (1), *Peak MV Flow* (2), and *LA Systole* (3). Top-right of each panel shows the quantification of each classification of particle as a percentage, and Bottom-right shows the MV flow wave form with the instant of each timepoint under consideration. The top panel shows the Pre CA results when the patient exhibited AF while the bottom shows the post CA results when the patient was in SR.

**Figure 9:**
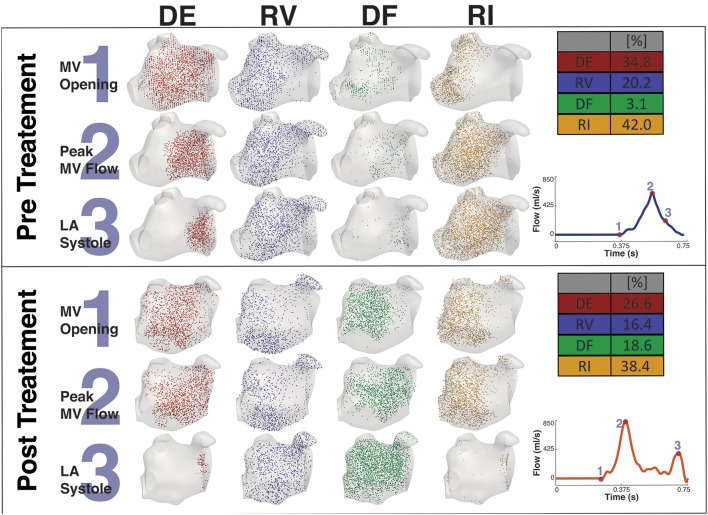
Particle tracking results Patient B. The image shows the particle tracking results for patient B. The image consists of two panels. Each panel shows the visualization of the different classification of particles DE, RV, DF, and RI at three points in the cardiac cycle, just prior to *MV Opening* (1), *Peak MV Flow* (2) and *LA Systole* (3). Top-right of each panel shows the quantification of each classification of particle as a percentage, and Bottom-right shows the MV flow wave form with the instant of each timepoint under consideration. The top panel shows the Pre CA results when the patient exhibited AF while the bottom panel shows the post CA results when the patient was in SR.

Figures 8, 9 shows the particle tracking results for patient A and B respectively. Each figure shows the results pre and post CA treatment. The results are highlighted at three time points as described previously, the instant prior of the MV opening, the instant of peak MV flow, and the instant of LA systole associated with the booster pump function of the LA. The figure also contains a table of the percentage breakdown of each class of particles with a color key for each class. The relevant MV flow waveform is also provided at the bottom right of each panel. Table 2 provides a comprehensive quantitative breakdown of the proportion of particles attributable to each class, including a breakdown of which DF and RI particles are attributable to the superior and inferior Left Pulmonary Vein (LPV) and Right Pulmonary Vein (RPV). In both patients a decrease in RV was observed after CA (24.7 vs. 7.7% in patient A and 20.2 vs. 16.4% in patient B, pre- and post-CA respectively). This change was associated with a substantial increase in DF post-treatment (1.9 vs. 15.3% in patient A and 3.1 vs. 18.6% in patient B). The combined RV and RI pre-CA was 65.9 and 43.3% for patients A and B respectively, and 62.2 vs. 54.8% post-CA.

**Table 2:** Comparative breakdown of proportion of particle classification.

**Particle**	**Patient A**	**Patient A**	**Patient B**	**Patient B**
**classification**	**Pre CA (%)**	**Post CA (%)**	**Pre CA (%)**	**Post CA (%)**
Delayed Ejection (DE)	32.2	35.3	34.8	26.6
Residual Volume (RV)	24.7	7.7	20.2	16.4
Direct Flow (DF) superior LPV	0.9	2.0	0.1	4.3
Direct Flow (DF) inferior LPV	0.2	4.3	0.3	4.1
Direct Flow (DF) superior RPV	0.6	7.5	1.9	7.7
Direct Flow (DF) inferior RPV	0.2	1.6	0.8	2.4
Direct Flow (DF) Total	1.9	15.3	3.1	18.6
Retained Inflow (RI) superior LPV	3.7	6.0	1.9	5.4
Retained Inflow (RI) inferior LPV	21.3	12.2	11.7	4.9
Retained Inflow (RI) superior RPV	21.3	13.3	25.1	22.7
Retained Inflow (RI) inferior RPV	11.9	4.2	3.4	5.4
Retained Inflow (RI) Total	41.2	35.6	42.0	38.4

*The table provides a comprehensive breakdown of the proportion of particles which can be attributed to each particle classification, including a breakdown of the proportion of DF and RI particles associated with the superior and inferior LPV and RPV*.

### 3.4. Thermal Analysis

**Figure 10:**
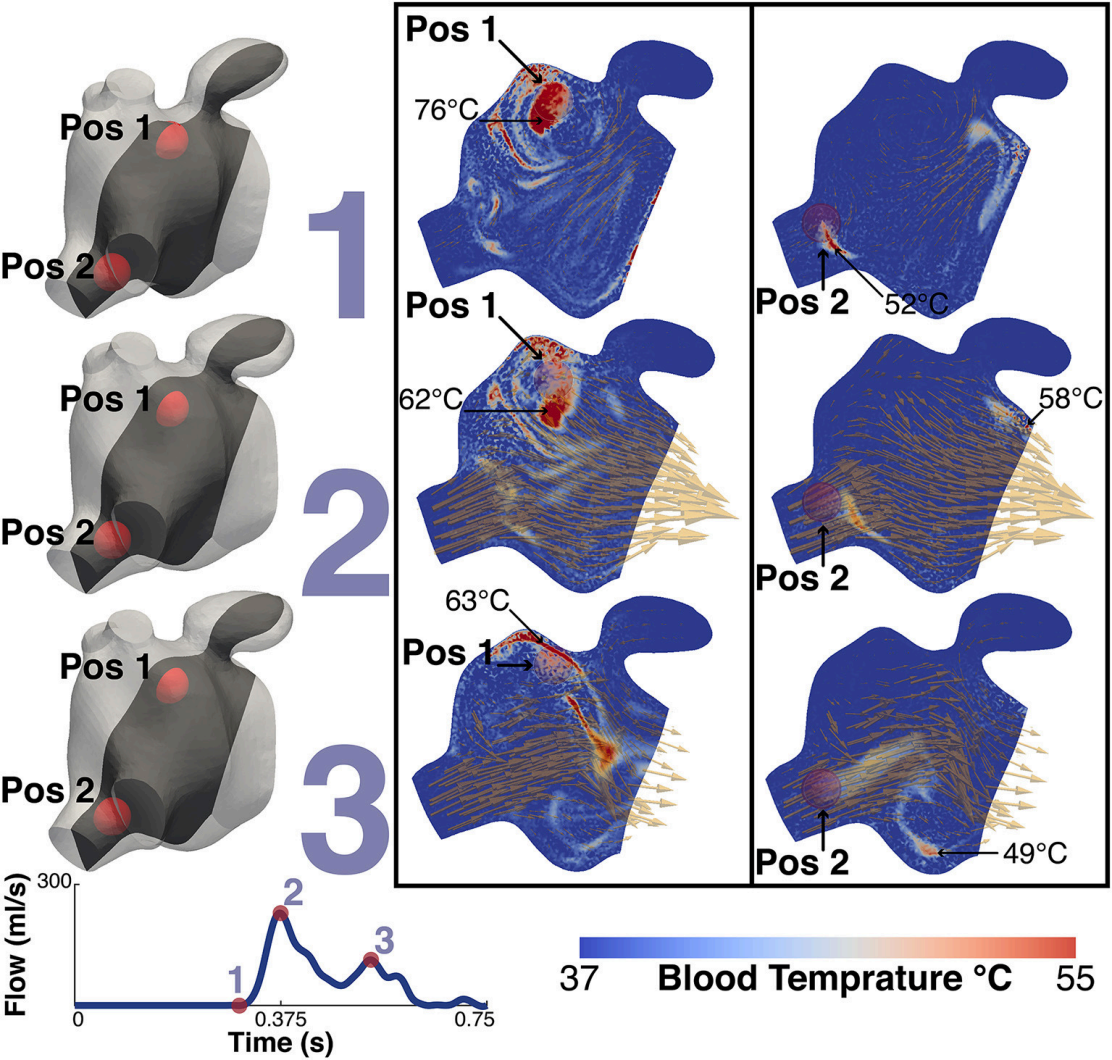
Thermal simulation results. The image shows the results of the thermal simulation for patient A after 5 cycles. The results are highlighed at three points in the cardiac cycle, just prior to *MV Opening* (1), *Peak MV Flow* (2) and *LA Systole* (3). The catheter tip was placed in two locations and these are represented in the two panels. The left shows the results when the tip is in the vicinity of the LAA (Pos 1) while the right shows the results when the tip is in the vicinity of the higher velocities of the inferior RPV. The two positions are indicated on each plot. The maximum temperature and the location are also indicated. For illustrative purposes the instantaneous velocity field is also visualized as orange arrows. To the left of the panels are volume representations of the slice location and the relative locations of the two tip positions. Bottom left shows the MV flow waveform.

Figure 10 shows the thermal simulation results for Patient A. The simulation was run with the catheter tip in two locations, Pos 1, with the catheter tip in the vicinity of the LAA, and Pos 2, with the catheter tip in the vicinity of the inferior RPV. The figure features a slice through the volume intersecting with the inferior RPV, the two tip locations, the LAA and the MV. For reference the figure also shows the velocity field overlaid on the thermal results. To the right of the thermal results is a volume representation of the slice orientation and tip locations. The figure also describes the maximum temperatures and their locations at that instant in the cycle.

The temperature of the catheter tip was set to 90°C, although this temperature was never actually recorded in the blood as the heat was advected immediately away from the tip. At the time point prior to the MV opening, the maximum temperature at the catheter tip was approximately 83°C and the maximum blood temperature was 76°C for Pos 1 and 58°C for Pos 2. When the tip was in Pos 1, close to the LAA, the maximum temperature observed in the LAA was 47°C. When in Pos 2, the temperature in the LAA did not exceed 40°C.

## 4. Discussion

This study performed thermal-fluid simulations in two patients who presented AF before treatement and were brought back in SR following CA. The results highlight significant differences in the LA fluid dynamics. In both cases flow velocities during AF were severely compromised and the vortex formation dynamics were also altered, in agreement with previous patient measurements based on the application of 4D flow MRI and phase-contrast MRI (Fluckiger et al., 2013; Masci et al., 2017a,b). The most noticeable difference between the two states was observed in the marked decrease during AF of the atrial booster pump function, which is responsible for the A-wave at the end of ventricular filling (atrial kick). This function was almost entirely absent in patient B, in analogy with previous findings that showed that suppression of atrial kick during AF can lead to a decrease in the total blood volume ejected into the LV by approximately 10% alone (Koizumi et al., 2015).

Lower blood velocities also corresponded to a decrease in the kinetic energy and viscous dissipation rate integrated over the total cavity volume. If this is to be expected given the direct proportionality with flow velocity, the results also showed that the peak kinetic energy and viscous energy dissipation during AF occurs earlier in patient A and later in patient B. This discrepancy is related to the fusion of the MV flow peak velocities into a single peak that occurs later in diastole for patient B, which does not occur in patient A, where the double-peaked flow is retained in AF (Figures 4, 5). This shows that the main contribution to the kinetic energy is during the mitral E-wave as expected from the mitral flow profile. However, in the fused peak case, the E-wave is delayed and the A-wave suppressed in the fusion, supporting the observation that the atrial booster pump function is the most compromised in the fibrillating atrium. The fusion of the two flow peaks at the MV is also observed in cases of severely impaired LV relaxation, where a slow fall in the LV pressure leads to a longer isovolumic relaxation time and to a reduction in the E-wave.

The main consequence of this impaired atrial contraction is a sharp increase in the proportion of flow that resides in the LA for more than one cycle. During AF, the sum of the RV and RI flow components is 65.9% in patient A and 64.2% in patient B, which is successfully reduced to 44.7 and 54.8% after restoration of SR, in line with the results obtained in a recent study of the healthy LA (Gaeta et al., 2018). Note that the majority of these slow particles (RV+RI) are located in the LAA, which is a well-known site for thrombus formation in AF.

In both patients, restoration of SR leads to an increase in the diastolic DF that is particularly evident in the RPVs (in particular in the superior RPV). According to a PC-MRI based study, the right side of the pulmonary veins provides inflow that is conveyed along the atrial wall to the MV with minimal entrainment into the systolic vortex in the middle of the atrial cavity (Fyrenius, 2001). This increase in the diastolic DF from these veins post-treatment suggests therefore an improvement in conduit function. Supporting this conclusion, in patient A this increase in diastolic DF from the right side is also associated with a marked decrease in the RI component from the RPVs post-ablation.

Interestingly, while RI is mainly located near the PV side pre-treatment, LAA is predominantly occupied by RV flow, which has the highest residence time in the LA. This is particularly relevant when localized heating during CA is delivered in a position close to the opening of the appendage, such as Position 1 in the simulations. This scenario sees an increase in the temperature of the RV flow in the appendage, which reaches 47°C. This value is in the range of the threshold of 50°–80°C for triggering plasma aggregation in stationary blood (Demolin et al., 2002). This critical value is however exceed in the blood close to the tip of the catheter during the procedure. When ablation is performed at the level of the pulmonary vein the blood temperature does not rise as much, suggesting that the continuous inflow plays an important role in heat advection.

It is worth noticing that the DF flow component reported here represents the blood that enters the LA during diastole and leaves it in the following systole. Given that the PVs do not have valves, the inflow to the LA is continuous and occurs during both systole and diastole. Our current workflow does not take into account the flow that enters the LA during systole and therefore this additional contribution is not included in DF. This however does not detract from the observation that CA has a substantial beneficial effect on the DF flow components, which increases after restoration of SR.

Albeit the recognized importance of blood hemodynamics in the left ventricle as a herald of structural adaptive mechanisms (Pedrizzetti et al., 2014, 2015; Mittal et al., 2016), the LA hemodynamics has received comparatively less attention. In this context, the present study aims to demonstrate the value of image-based blood flow modeling as a predictive tool that is not restricted to a single application, and can be applied to improve CA and provide insights into the mechanisms underlying AF pathophysiology and leading to life-threatening complications, such as subclinical thromboembolism.

## 5. Conclusions

This study presents novel patient-specific modeling workflow for characterizing the thermal-fluid dynamics in the atria of SR and AF patients, post- and pre-CA, respectively. The models enable simulation of atrial blood flow and its changes from SR to AF, including: (1) substantial decrease in the average flow velocity and kinetic energy, as well as increase in the percentage of blood remaining in the LA for longer than one cycle after CA, (2) the emergence of regions of blood stasis in the LAA and the resultant increase of blood temperature during CA in this region, which reaches values close to the threshold for coagulation. Decreased blood flow velocities during AF, region of blood stasis and the localized heating of the blood surrounding the CA sites all have been associated with increased risks of thrombus formation. Therefore, the novel workflow can be utilized for the improved quantification of the risks of thromboembolism and stroke, especially in challenging patient populations with low empirical CHADS2VaSc score.

## Author Contributions

DD-M: main contributor; DM, BR, AQ, HC, EK, and MO: support; DN, OA, and AdV: support contributions; MO, DN, OA, and AdV: senior authors.

### Conflict of Interest Statement

The authors declare that the research was conducted in the absence of any commercial or financial relationships that could be construed as a potential conflict of interest.

## Supplementary Material

Supplementary Video 1 Vortices in SR: The video shows the velocity magnitude field overlaid with red velocity vectors on a plane cut intersecting the MV, RPV, LPV and the LAA for patient A in SR. The video shows multiple fast moving, short-lived vortices created at peak systole, one is seen between the LPV and RPV (left) and another one near the LAA (top right). This is typical of patients in SR.

Supplementary Video 2 Vortices in AF: The video shows the velocity magnitude field overlaid with red velocity vectors on a plane cut intersecting the MV, RPV, LPV and the LAA for patient A in AF. The video shows a single relatively slow vortex moving anti-clockwise in the middle of the LA cavity.
